# Genotyping of Multiple Clinical Samples with a Combined Direct PCR and Magnetic Lateral Flow Assay

**DOI:** 10.1016/j.isci.2018.09.005

**Published:** 2018-09-08

**Authors:** Chao Zhang, Xiaonan Liu, Yao Yao, Kewu Liu, Wenli Hui, Juanli Zhu, Yaling Dou, Kai Hua, Mingli Peng, Zuankai Wang, Alphonsus J.M. Vermorken, Yali Cui

**Affiliations:** 1 College of Life Sciences, Northwest University, Xi'an, China; 2 Shaanxi Provincial Engineering Research Center of Nano-Biomedical Detection, Xi'an, China; 3 Department of Clinical Laboratory, Peking Union Medical College Hospital, Chinese Academy of Medical Sciences, Beijing, China; 4 Department of Mechanical Engineering, City University of Hong Kong, Hong Kong, China

**Keywords:** Clinical Genetics, Analytical Chemistry, Fluidics, Biochemical Analysis

## Abstract

Developing a sensitive, low-cost, and easy-to-use point-of-care testing system for genotyping is important for informing treatment decisions and predicting the risk of underlying diseases. Conventional methods normally require complex operational procedures as well as expensive and sophisticated instruments. Here, we report a general approach that enables us to detect the genotype of multiple sample types directly without DNA purification. Moreover, the PCR results can be further quantitatively analyzed based on a magnetic lateral flow assay (MLFA) system, which avoids multiple steps needed for conventional nucleic acid biosensors. As a demonstration, we show that three genotypes of aldehyde dehydrogenase 2 (*ALDH2*) can be identified using a small volume of sample with an accuracy of 100% and a sensitivity of 1.0 × 10^2^ cells/μL, which are better than those of the gold standard methods. We believe that the direct PCR-MLFA system represents a significant advance toward the development of portable, sensitive biomedical platforms.

## Highlights

• Multiple samples (blood, DBS, saliva and buccal swab) for PCR template directly
• Combined direct PCR and magnetic lateral flow assay for *ALDH2* genotyping
• Genotypes can be obtained within 90 min

## Graphical Abstract

**Figure:**
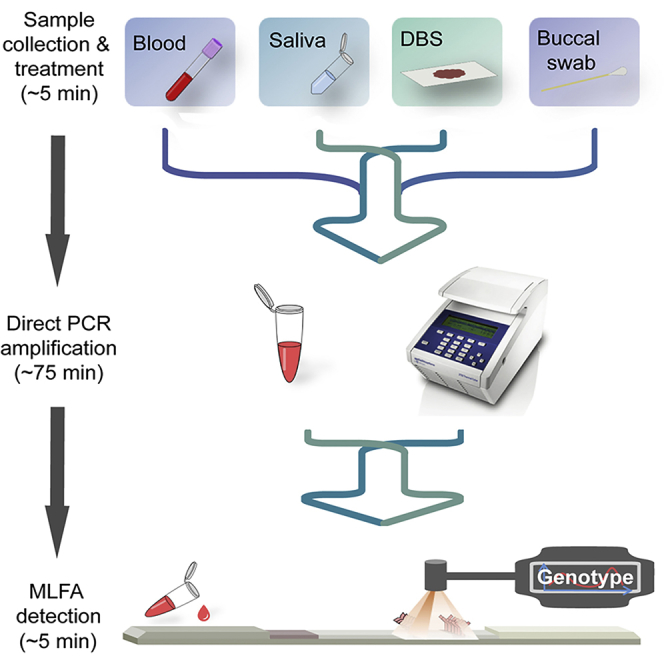

## Introduction

SNPs represent the most frequent type of variation (approximately 90%) in the human genome (Wang et al., 1998, Ye et al., 2001), the detection of which is of great significance to association studies of complex diseases (Ngo et al., 2016), pharmacogenomics (Li et al., 2015), population genetics (Liu and Fu, 2015, Mccarroll et al., 2008), and physical mapping (Charlier et al., 2008). There are three possible genotypes for each biallelic SNP locus, wild-type and heterozygous and homozygous mutation types, which correspond to different phenotypes and lead to different clinical manifestations. For genotyping, three distinct operational steps are typically required: sample preparation, target amplification, and signal readout. Although each step can be considered individually, it is important to emphasize that a key challenge for the development of such nucleic acid detection methods is streamlining the steps as much as possible. Existing methods usually require sample purification via a labor-intensive, time-consuming, and expensive sample preparation step due to the stringent requirements for DNA quality and quantity (Aljanabi and Martinez, 1997, Golenberg et al., 1996), which also enhances the risk of cross-contamination between samples (Brouwershaven et al., 2010, Kelly and Woolley, 2005). Nevertheless, the amplification products are usually analyzed by agarose gel electrophoresis, a further step such as hybridization, or professional software in a signal readout step. Therefore, a universal platform that can visually or automatically read out the PCR results is lacking. Moreover, existing analytical methods are usually too slow to support immediate treatment decisions or to be implemented as point-of-care diagnostics (Schoepp et al., 2017). Scientists have developed various methods to increase the timeliness, sensitivity, and specificity of genotyping using more sophisticated techniques and instruments. Currently, DNA sequencing is considered to be the gold standard for laboratory-based SNP detection (Hawkins, 2017); however, high operating expenses and high-precision equipment requirements have impeded its wide application, particularly in resource-poor settings.

Establishing a genotyping system that is adapted to a wide variety of sample types and avoids DNA isolation is challenging because current strategies for genotyping based on PCR require relatively high purity of DNA. However, samples commonly used in clinical settings, such as blood, saliva, dried blood spot (DBS), and buccal swab, are not easy to genotype without DNA purification because cell membranes prevent cell damage and DNA release. Thus, developing a general strategy for a genotyping system that analyzes multiple sample types without DNA isolation has remained elusive. Although several protocols and commercial kits that perform PCR amplification without DNA extraction from whole blood (Nanayakkara et al., 2017) and saliva (Ambers et al., 2018) have been developed, they still require complex reagents, large volume of samples, and substantial power consumption.

To overcome this challenge, we have established a new approach that makes direct PCR feasible by leveraging a simple process that enables the efficient release of nucleic acids and inactivates the natural PCR inhibitors in a sample. Many methods have offered sensitive and specific approaches for genotyping. However, the tremendous potential of gene detection in clinical practice has been limited thus far by multiple time-consuming steps and the need for professionally trained staff and costly equipment to obtain genotyping results. Furthermore, the results are usually analyzed by agarose gel electrophoresis (most PCR-based methods) (Ramire-Zexpósito et al., 2016, Wu et al., 2015), a further reaction step, and professional software (e.g., biochip, sequencing, and mass array) (Nijveen et al., 2013, Xu et al., 2012, Yi et al., 2014). To address these issues, we used a magnetic lateral flow assay (MLFA) system to interpret the results through visualization or a magnetic signal reader. The MLFA is derived from traditional lateral flow strips using our own synthetic gold magnetic nanoparticles (GMNPs) as labels, which can be harnessed to develop simple and portable devices that enable both the visual and quantitative interpretation of data for point-of-care SNP detection.

In this study, we developed an integrated strategy for miniaturizing simple process laboratory assays to shorten their complex steps and demonstrated that the entire contiguous sample-to-answer workflow could enable the genotyping of a clinical sample in less than 90 min. We combined two improved technologies into one system: a direct PCR assay for DNA amplification without purification and an MLFA system to read the results rapidly and automatically. These contributions address several bottlenecks of current methods while providing the advantages of simplicity, cost, portability, and quantitative genotyping. This technology is one step closer to realizing the ubiquitous availability of gene tests, which can ultimately aid rapid medical decisions.

## Results

### Direct PCR-MLFA System Design

**Figure 1:**
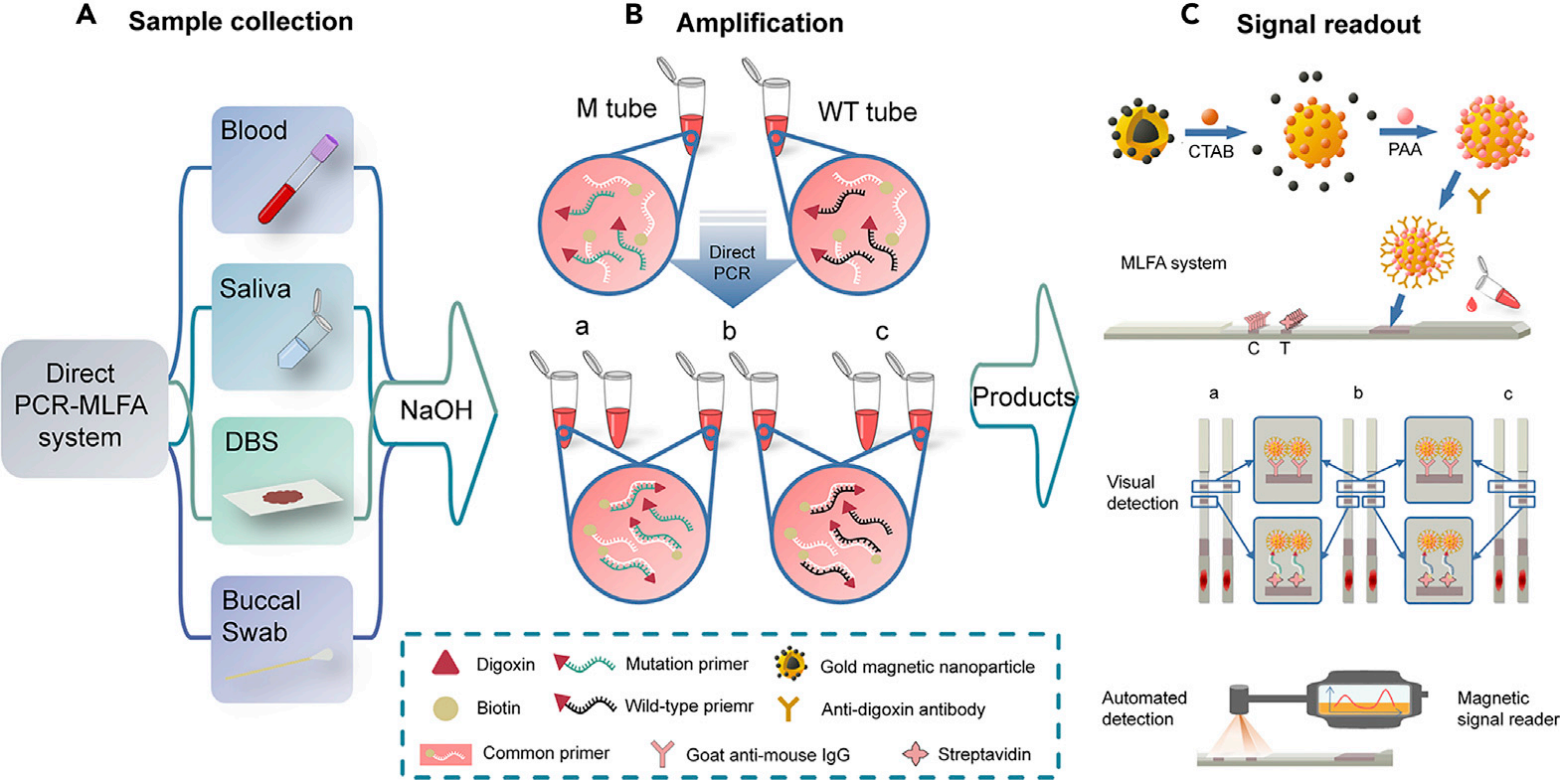
Schematic Diagram of the Direct PCR-MLFA System (A) Sample collection and treatment with NaOH. (B) Target amplification using specifically designed primers (corresponding to M and WT). (C) Signal readout: the composition and working principle of the magnetic lateral flow assay. When the amplification products are added to the MLFA strip, they are driven forward by capillary action, and magnetic signals and color appear at the control line (C line) or both the control and test lines (T line). The results can be read visually, or they can be detected automatically with a magnetic signal reader.

The proof-of-concept scheme described in this article uses direct PCR followed by an MLFA (direct PCR-MLFA) for SNP detection in two steps: target amplification and signal readout. Figure 1A shows the collection and treatment of clinical samples. Designed as a universal genotyping platform, the direct PCR-MLFA system could be used for a variety of sample types. Four frequently used clinical samples, including whole blood, DBS, buccal swab, and saliva, were selected and successfully applied to the established platform, which indicates that the direct PCR-MLFA system is a platform with general applications that could be applied to type other genes and sample types. The collected sample is treated with NaOH solution followed by direct PCR amplification with specifically designed primers as shown in Figures 1B and S1 and Table S1. For convenience, we refer to the mutation type as “M” and the wild-type as “WT.” For each sample to be analyzed, two separate reactions (M tube and WT tube) are run simultaneously using the same treated sample. Two sets of allele-specific primers (for the WT and M sequences) are added to two different tubes for amplification, and target PCR products are acquired only when the 3′ end of the specific primer is complementary with the template. The direct PCR procedure takes only about 5 min for sample collection and treatment and about 75 min for amplification. The three possible outcomes are presented in Figure 1B: (a) homozygous mutation type, (b) heterozygous mutation type, and (c) wild-type.

As shown in Figure 1C (signal readout step), the MLFA is performed using a strip that is composed of five components, a sample pad, a conjugate pad, a nitrocellulose membrane, an absorbent pad, and a plastic cushion. Amplicons are added to the sample pad and then read automatically with a magnetic reader or visualized with the unaided eye. The PCR products added to the sample pads of the MLFA strips can be combined with anti-digoxin (Dig) antibody-conjugated GMNPs (GMNPs-anti-Dig) on the conjugate pad due to the specific combination of a Dig label at the 5′-end of the specific primers and an anti-Dig antibody. As the products migrate along the strip, the PCR product-GMNP-anti-Dig conjugates can be captured by streptavidin immobilized at the test line (T line) due to the biotin label at the 5′ end of the common primers, which produces a red band at the T line. The rest of the GMNPs-anti-Dig keep moving and are captured by goat anti-mouse IgG at the control line (C line), which forms another red band that confirms the efficacy of the MLFA system. In the absence of target PCR products, no red band is observed at the T line. The results can be obtained using a magnetic reader to detect the magnetic signals at the T and C lines, or they can be read visually according to the presence of a red band at the T line. The MLFA system operating procedure can be completed within 5 min from amplicon loading to result readout.

The assay control system must be capable of verifying the amplification step and testing the lateral flow step. For the amplification step, only when at least one of the two test strips has a red band on the test line after lateral flow detection the amplification test is treated as valid. This is because one SNP can only have one of the three genotypes: wild-type, heterozygous mutation type, and homozygous mutation type. Therefore, the dual strips themselves can be applied as a control to ensure that the amplification process is effective. For the signal readout step, the control line is included for testing the GMNPs and the proper reagent flow in each strip. Meanwhile, a positive control using sequenced genomic DNA (wild-type and homozygous type) as templates and a negative control using NaOH-treated double-distilled water (ddH_2_O) as template are performed simultaneously. To sum up, both amplification and lateral flow procedure are under appropriate control.

### Biological Sample Treatment

Technically, the factors that cause challenges for genotyping clinical samples directly include the enclosure of nucleic acids by a membrane and the presence of commensal or contaminating organisms in the clinical samples, which can inhibit PCR amplification. If the sample treatment method does not lyse the membrane or inactivate the inhibitors, then the genotype cannot be determined accurately. To facilitate the release of DNA from cells, various methods have been applied, such as using expensive special materials or complex solvents (Gong and Li, 2014, Saavedra-Matiz et al., 2013). Despite their efficacy, the utility of these procedures is questionable. Therefore, the search for an inexpensive and nontoxic procedure is ongoing. Herein, we present a simple, inexpensive yet powerful chemical procedure devoid of costly materials and complex solutions for DNA lysis. Briefly, NaOH solution was used for cell lysis, which allowed rapid DNA release from the collected samples.

**Figure 2:**
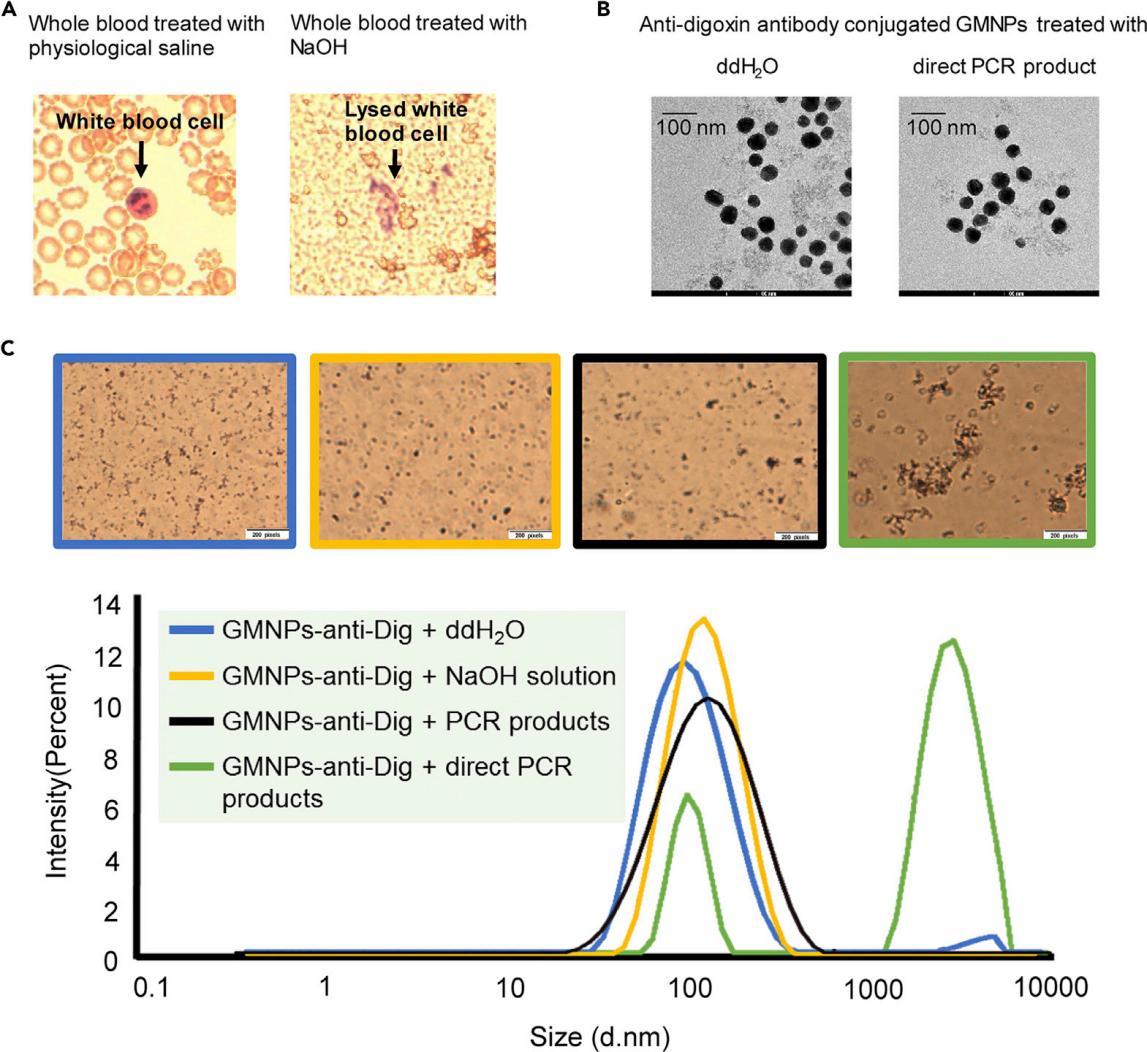
Effect of NaOH in the Direct PCR-MLFA System (A) Image of blood cells treated with physiological saline or NaOH. (B) Transmission electron microscopy images of GMNPs-anti-digoxin antibodies treated with ddH2O or direct PCR product. (C) Images and average size (diameter) of GMNPs-anti-digoxin antibodies under different treatment conditions.

First, we tested the effects of the NaOH solution on cells in the samples. As shown in Figure 2A, a large amount of cellular debris was found in the whole blood samples treated with NaOH, whereas the cellular integrity of the blood cells was preserved in physiological saline. Similar results were found with saliva and buccal swab solutions treated with NaOH and physiological saline. The results indicated that cell lysis occurs during alkaline treatment, releasing DNA from the cells into the sample solution. The success of direct PCR amplification may be affected by the sample dilution factor, which tends to significantly reduce the concentration of PCR inhibitors present in the sample. We have failed to obtain any amplification products in NaOH-based direct PCR at a 1:1 dilution, suggesting that dilution may play a critical role in the success of direct PCR.

As the pH of the NaOH solution can be as high as 13.0, we next evaluated the pH value of the reaction system, which affects the PCR efficiency of the target amplification step as the optimal working pH for Taq DNA polymerase is 7.4–8.3, according to Min et al. (Min et al., 2005). We tested the pH value at each stage of the direct PCR process after NaOH addition. As shown in Figure S2, the pH value of the NaOH solution was 13.0; when the NaOH solution was added to the sample, the pH value of the mixture was 11.8–12.0. However, the pH value drops dramatically to 7.6–8.1 after mixing the NaOH-treated sample with the PCR reaction buffer, and no significant difference in pH value was observed between the PCR reaction buffer with and without the NaOH-treated sample. Thus, the Tris-HCl buffer in the PCR reaction played a determinant role in pH control and provided an optimal working environment for DNA polymerase.

### Direct PCR Amplification

Our next goal was to optimize the direct PCR procedure to improve the specificity while eliminating background noise (non-specific amplification or primer dimerization). We designed allele-specific primer pairs and optimized their concentrations in the direct PCR system. By testing various conditions (100, 75, 50, 25, and 10 nM) simultaneously, the optimized primer concentrations were determined to be 50 nM for each primer (Figure S3A). In the current study, to assure specificity, the allele-specific primers were designed to contain an additional mismatch at the penultimate 3′ nucleotide to increase the strength of allelic discrimination according to the principle of amplification-refractory mutation system (Little, 1995, Newton et al., 1989). Moreover, we also studied the effect of sample concentration on the direct PCR-MLFA system signal. The response signal (T line) increased when the sample solution in the PCR mix was raised from 3 to 5 μL but then leveled off, indicating that the optimal volume was attained (Figure S3B).

To assess the ability of the established direct PCR to eliminate false-positive results due to carryover contaminants during SNP detection, we carried out contamination prevention reactions using dUTP and uracil-DNA glycosylase (UDG). Using dUTPs during amplification and pretreating subsequent samples with UDG to remove carryover PCR products from prior reactions are common practices (Laird, 2010). To verify the effectiveness of the UDG system in the direct PCR-MLFA system, the sample solution and/or a few previous PCR products were combined into tubes as template before direct PCR amplification, and a digestion step (2 min at 50°C) was executed. The UDG enzyme specifically cleaves uracil bases from any uracil-containing PCR products. As a result, the carryover contaminants generated from previous PCR reactions are effectively eliminated (as shown in Figure S3C), and they cannot be used as templates for re-amplification. Hence, UDG is able to prevent the amplification of carryover contaminant templates, which significantly decreases the likelihood of false-positive results during direct PCR-MLFA analysis.

Once the direct PCR products are loaded onto the sample pad of the MLFA strip, the stability of the GMNPs-anti-Dig is invariably challenging for lateral flow. To verify the morphological stability of the GMNPs-anti-Dig, transmission electron microscopic (TEM, Figure 2B) images were observed. As shown in the TEM image, the GMNPs-anti-Dig were well dispersed in ddH_2_O and the direct PCR product solution had no effect on the GMNPs-anti-Dig. The good dispersion of the GMNPs-anti-Dig was further confirmed by measuring their average size (diameter) under different conditions (Figure 2C). A single, sharp peak at 100–150 nm was observed with GMNPs-anti-Dig in ddH_2_O (blue), NaOH solution (yellow), and PCR product solution (with purified DNA as template; black). However, in the direct PCR product solution with NaOH-treated sample, there are two peaks at 100 and 3,100+ nm (green), likely due to the cellular debris in the treated sample.

When direct PCR-MLFA was performed with collected samples, the best results were obtained at a final reaction volume of 50 μL, which contained 10× PCR buffer, 0.2 mM of each dNTP (dATP, dUTP, dCTP, and dGTP), 3 mM of MgCl_2_, 0.5 U of HotMaster Taq DNA polymerase (TIANGEN Biotech Co., Ltd., Beijing, China), 0.5 U of UDG (Shanghai ShineGene Molecular Biotechnology Co., Ltd., Shanghai, China), 50 nM of forward and reverse primers (forward M primer in M tube and forward WT primer in WT tube, reverse primer added in both tubes), and 5 μL of sample-treated solution as template. All the amplifications were performed according to the following parameters: two initial denaturation steps for 2 min at 50°C and 3 min at 94°C; 32 cycles of 5 s at 94°C, 10 s at 60°C, and 30 s at 65°C; and one step of 10 min at 65°C.

### Performance of the Direct PCR-MLFA System

**Figure 3:**
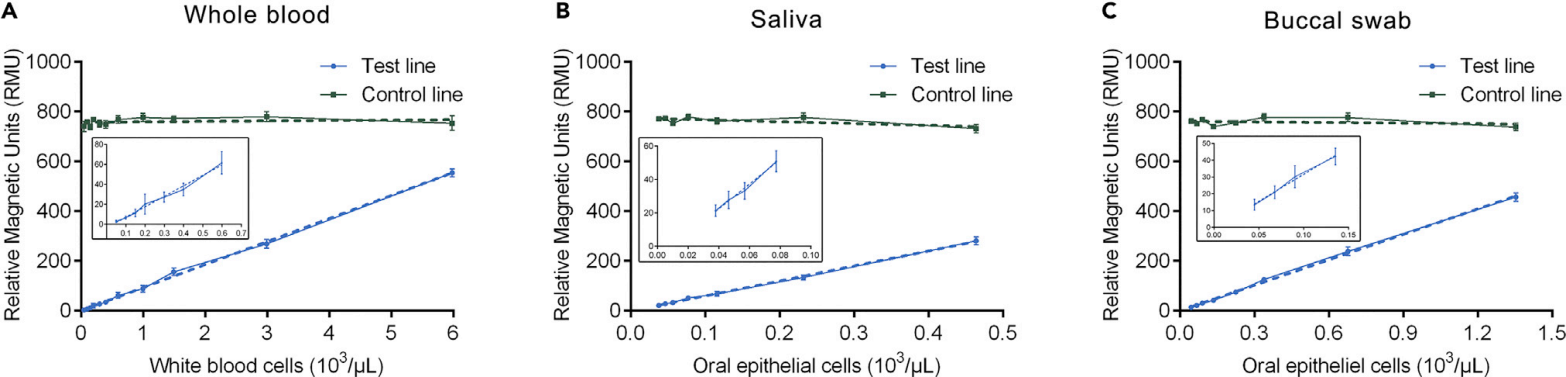
Standard Curve for the Direct PCR-MLFA Detection Standard curve for the detection of the average magnetic signal at the T line relative to the respective WBC count in whole blood (A) or the oral epithelial cell density in (B) saliva, and (C) buccal swab samples.

To test the detection limit of the direct PCR-MLFA system, we assayed serial dilutions of whole blood, saliva, and buccal swab samples using a magnetic signal reader to obtain accurate parameters. For the direct, quantitative measure of the limit of detection (LOD), serial dilutions of blood, saliva, and buccal swab samples were prepared in physiological saline and analyzed with the direct PCR-MLFA system. As shown in Figure S4, whole-blood samples containing different densities of white blood cells (WBCs), ranging from 0.06 × 10^3^–5.96 × 10^3^ cells/μL (Figure S4A); saliva samples containing different densities of oral epithelial cells, ranging from 0.03 × 10^3^–0.46 × 10^3^ cells/μL (Figure S4C); and buccal swab samples containing different densities of oral epithelial cells, ranging from 0.04 × 10^3^–1.35 × 10^3^ cells/μL (Figure S4E), were examined with our system. The PCR amplification efficiency (relative magnetic units [RMUs]) significantly improved as the concentration of WBCs and oral epithelial cells increased. Figures S4B, S4D, and S4F show the magnetic signal peak value of the T line and C line at different concentrations of WBCs and oral epithelial cells, which simulated the output of the magnetic reader. Even when the dose was as low as 1.0 × 10^2^ cells/μL, the specificity of the test remained high, with no false-negative results. Normally, the number of WBCs in whole blood is in the range of 4.0 × 10^3^–10.0 × 10^3^ cells/μL. Therefore, the LOD of the direct PCR-MLFA system is fully applicable for clinical testing. Moreover, as shown in Figure 3, a standard curve for detection was plotted as the average magnetic signal at the T line relative to the respective concentrations of WBCs or oral epithelial cells. The high correlation coefficient (R^2^ > 0.997) indicated that the direct PCR-MLFA system could be applied for DNA quantification.

We further assessed the reproducibility of the direct PCR-MLFA system by analyzing amplicons with wild type, heterozygous mutation, and homozygous mutation genotypes (verified by DNA sequencing) of aldehyde dehydrogenase 2 (ALDH2*2). Three amplifications per sample were carried out, followed by visual detection using lateral flow strips. The three pairs of strips were prepared using three different batches of reagents under the same protocol. The results presented in Figure S5 indicated that no differences were detected among the three pairs of strips. The present study therefore illustrates the high reproducibility, sensitivity, and reliability of our direct PCR-MLFA system, providing a useful method for direct SNP detection in clinical samples.

### Evaluation of the Direct PCR-MLFA System Using Clinical Samples

**Figure 4:**
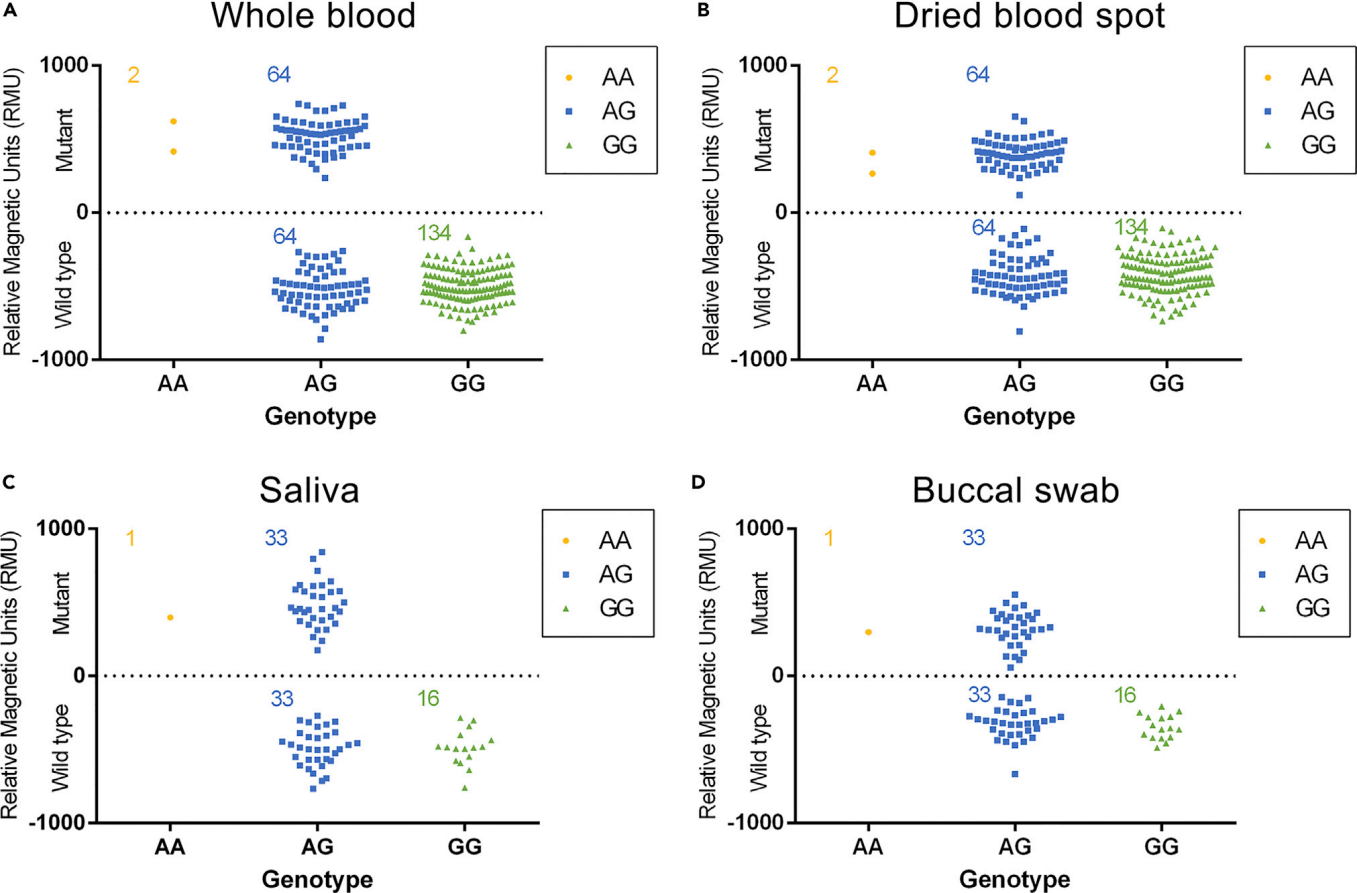
Genotyping Results for Multiple Sample Types (the Magnetic Signal Value of the Wild-Type Was Defined as a Negative Value) (A) Genotyping results of 200 whole blood samples. (B) Genotyping results of 200 dried blood spot samples. (C) Genotyping results of 50 saliva samples. (D) Genotyping results of 50 buccal swab samples.

Having established the direct PCR-MLFA system, we next tested its accuracy with clinical samples (matched 200 whole blood and 200 DBS samples; matched 50 buccal swab and 50 saliva samples). For each sample, the magnetic signal value (RMUs) of the T line was obtained. All blood samples were also sequenced by BGI (Beijing Genomic Institute, Beijing, China). As shown in Figure 4, the RMU of WT strip was defined as a negative value, and the M strip was defined as a positive value. Of the 200 whole blood samples and DBS samples, 2 samples had only an M RMU, 134 samples had only a WT RMU, and the remaining 64 samples had both M and WT RMUs (Figures 4A and 4B), indicating 2 homozygous mutation type samples, 134 wild-type samples, and 64 heterozygous mutation type samples among the 200 whole blood samples and DBS samples. Of the 50 saliva samples and buccal swab samples, 1 sample had only an M RMU, 16 samples had only a WT RMU, and the remaining 33 samples had both M and WT RMUs (Figures 4C and 4D), indicating 1 homozygous mutation type sample, 16 wild-type samples, and 33 heterozygous mutation type samples among the 50 saliva samples and buccal swab samples. No discrepancies were observed when the results of the direct PCR-MLFA system and DNA sequencing were compared (Table S2). The observed allele frequencies were 83% and 17% for *2G and *2A, respectively (calculated from *2G: F1 + F2/2 and *2A: F2/2 + F3). The frequencies of the ALDH2*2 allele measured with the direct PCR-MLFA system were not significantly different from those reported by Eng. et al. in the Chinese population (Eng et al., 2007). As shown in Table S3, the one-to-one correspondence between the genotyping results of the buccal swab and saliva samples using the direct PCR-MLFA system also indicated successful SNP detection using buccal swab and saliva samples.

### Limitation of Study

Here, we have established a sensitive, low cost, and easy-to-use SNP detection platform using the combination of the direct PCR and lateral flow assay, which enables us to obtain genotyping results within 90 min by using the clinically relevant samples (whole blood, DBS, buccal swab, and saliva) directly without DNA purification. It is envisaged that the direct PCR-MLFA system could be quickly adapted for the detection of other DNA mutation types, such as recombination, deletion, and insertion. Despite many advantages, it still remains elusive for us to detect the variation in the DNA copy number. Further efforts on the design of the amplification principle and primer sets will be explored to make the MLFA system applicable to copy number variation detection.

## Discussion

To date, most of the PCR-based genotyping methods rely on the use of the purified DNA, rather than the use of the clinical samples (Cavanaugh and Bathrick, 2018). Moreover, only a few conventional PCR-based genotyping methods are capable of achieving a sample-to-answer result directly from a clinical sample in less than 90 min (Broccanello et al., 2018, Di et al., 2017, White and Cantsilieris, 2017). Remarkably, Gomez-Martinez et al. have developed a multiplex linear-after-the-exponential (LATE)-PCR for visual detection of blood group genotype with turnaround time of approximately 1 hr by using a KAPA2G Fast HotStart DNA Polymerase (Gomez-Martinez et al., 2018). However, it is not clear whether this LATE-PCR method can be applicable to different sample types. By leveraging NaOH to release nucleic acids and inactivate the natural PCR inhibitors in a sample, our PCR-MLFA system is capable of genotyping target DNA with different clinical sample types directly by using a common Taq DNA polymerase without undergoing the conventional DNA isolation step. Thus, compared with LATE-PCR, which involves the use of genetically engineered HotStart DNA polymerase, our method is more affordable and versatile.

The treatment of samples with NaOH plays an important role in the direct PCR-MLFA system, making the whole testing procedure convenient and usable. First, compared with other SNP detection methods, the DNA purification step was eliminated in our assay with the help of NaOH treatment, which shortened the processing time from 1 or 2 hr to a mere 5 min. Second, by using NaOH-treated blood samples for PCR, the problem of cross-contamination, which exists in traditional blood DNA purification processes, is eliminated. Furthermore, various natural PCR inhibitors in the samples such as hemoglobin, IgG, lactoferrin, and proteases (Adams, 2005) are inactivated by NaOH treatment.

Lateral flow assay was chosen for signal readout because it is a well-established technique with several readout methods (Nayak et al., 2016, Sajid et al., 2015). By further leveraging on the GMNPs, the MLFA system was established, allowing for the qualitative analysis of genotype using an automatic magnetic reader. The major advantage of this assay is its rapid qualitative output of “Yes” or “No.” Compared with a previous study on SNP detection using PCR-based methods or DNA sequencing, our assay has significant advantages (Hui et al., 2016, Ngo et al., 2016, Yun et al., 2015, Zhang et al., 2016). Specifically, (1) existing genotyping methods based on PCR, which require purified DNA as template, such as PCR microarray, qPCR, and DNA sequencing, are time consuming because conventional DNA purification usually takes more than 1 hr, whereas the present assay system requires less than 5 min for sample preparation. Moreover, (2) the previously developed techniques usually require a complex operational procedure as well as expensive and sophisticated instruments that may not be available in many laboratories, whereas the present method provides an easy-to-operate and affordable on-site technique for genotyping with high efficiency. (3) The MLFA is ideal for quantification, because it is made with magnetic nanoparticles and there is no optical interfering with magnetic signal readout, which is distinct from traditional lateral flow assay.

Our MLFA chip provides an alternative to sequencing or real-time PCR for rapid genotyping while maintaining the advantages of lateral flow assay in terms of simplicity, affordability, and rapid qualitative/quantitative nucleic acid readout (Chen et al., 2016). For simplicity and affordability, the MLFA chip is designed with simple construction, materials, and regents and the cost is quite low and can be further reduced when mass-produced. Combined with direct PCR, we have shown that it is possible to perform PCR amplification using multiple sample types directly without DNA purification. This is significantly lower cost and faster readout than conventional genotyping methods.

Human *ALDH2*, which is responsible for the oxidation of aldehydes in the liver, was utilized as a model for SNP detection. Differences in *ALDH2* expression may contribute to a wide variety of human diseases, including cardiovascular disease, diabetes, and cancer (Chen et al., 2014). In addition, genetic polymorphisms of *ALDH2* alter susceptibility to ethanol intake as well as the risk of alcoholism and alcoholic complications, and *ALDH2* may possess important therapeutic potential against alcoholism and other forms of myocardial damage (Hou et al., 2017). The application of this rapid and direct PCR-MLFA system in additional SNP studies is an important next step that, if successful, would further validate and demonstrate its clinical utility. In addition, the standard curve obtained in this study had a high correlation coefficient, indicating that the direct PCR-MLFA assay could also be applied for human DNA quantification. Therefore, the assay could be used to determine trace amounts of DNA of interest among abundant background DNA, such as specific mutation detection in circulating tumor DNA (Thierry et al., 2014), and for complex gene quantification, both of which are clinically valuable (Gonçalves et al., 2016, Sidstedt et al., 2017).

## Methods

All methods can be found in the accompanying Transparent Methods supplemental file.

## Supplemental Information

Document S1. Transparent Methods, Figures S1–S5, and Tables S1–S3
