# Supplementary material for: Genotyping of Multiple Clinical Samples with a Combined Direct PCR and Magnetic Lateral Flow Assay
Source: iScience. 2018 Sep 8;7:170–9. doi: 10.1016/j.isci.2018.09.005 (PMC6153416; doi:10.1016/j.isci.2018.09.005)
Supplement: Document S1. Transparent Methods, Figures S1–S5, and Tables S1–S3 [file mmc1.pdf]

ISCI, Volume 7

Supplemental Information

Genotyping of Multiple Clinical Samples with a Combined Direct PCR and Magnetic Lateral Flow Assay

Chao Zhang, Xiaonan Liu, Yao Yao, Kewu Liu, Wenli Hui, Juanli Zhu, Yaling Dou, Kai Hua, Mingli Peng, Zuankai Wang, Alphonsus J.M. Vermorken, and Yali Cui

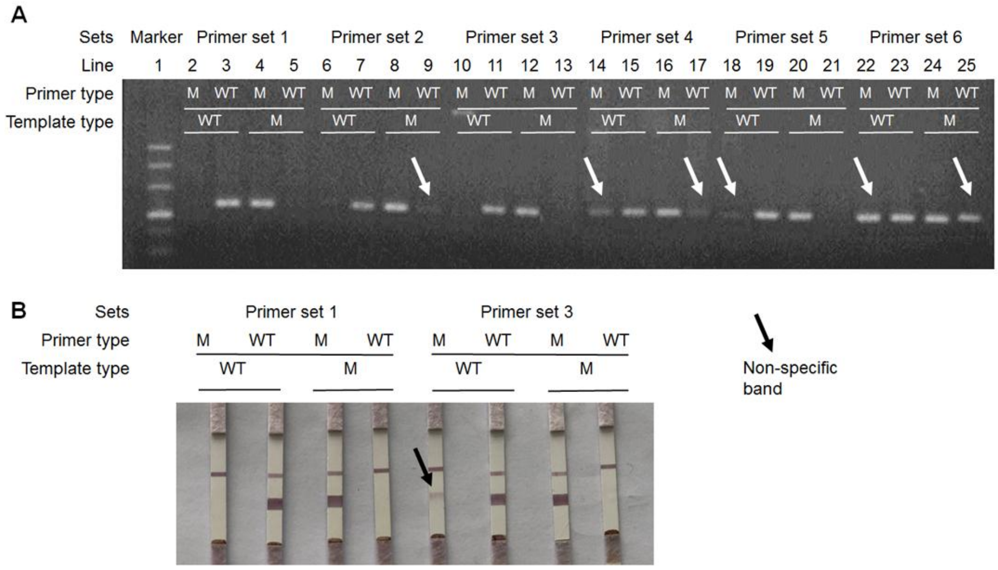

Figure S1, Related to Figure 1. Optimal primer set selection. (M=Mutant, WT=Wild Type)

(A) The primer sets were designed according to the principle of Amplification Refractory Mutation System (ARMS). We tested six sets of mismatched primers (as showed in Table S1) by agarose gel to select for the most specific primer sets. As showed in the figure, the results indicated that, except for primer set 1 and set 3, the other groups result in non-specific bands on the agarose gel.

(B) When the priemr set 1 and primer set 3 were applied to the lateral flow strip, the mutant primer of the primer set 3 showed a non-specific band.

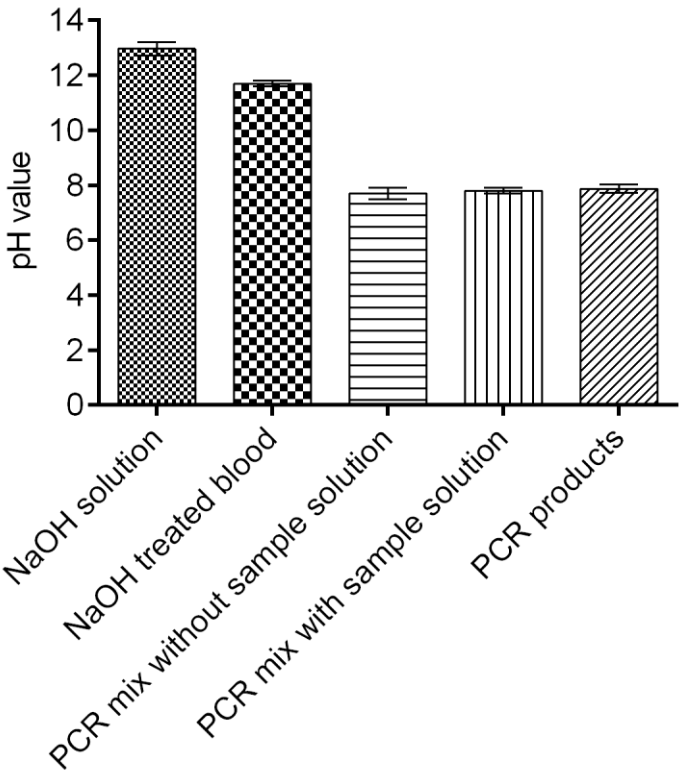

Figure S2, Related to Figure 2. The pH value at each stage of direct PCR assay.

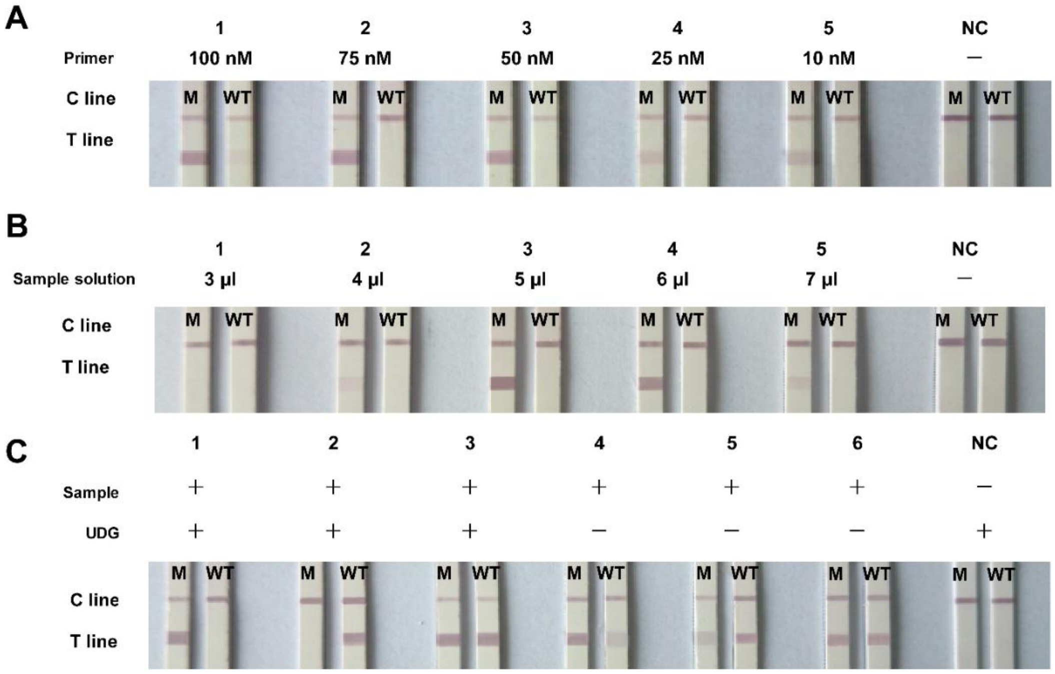

Figure S3, Related to Figure 2. Optimization of the direct PCR-MLFA system (M=M tube, WT=WT tube, NC=negative control, using NaOH treated ddH$_2$O as template instead of NaOH treated sample).

(A) Optimized primer concentrations were determined to be 50 nM.

(B) Optimized sample solution amounts were determined to be 5 μL.

(C) The effect of UDG used to prevent contamination. Line 1 and line 4: homozygous mutation sample; line 2 and line 5: homozygous wild type sample: line 3 and line 6: heterozygous mutation sample.

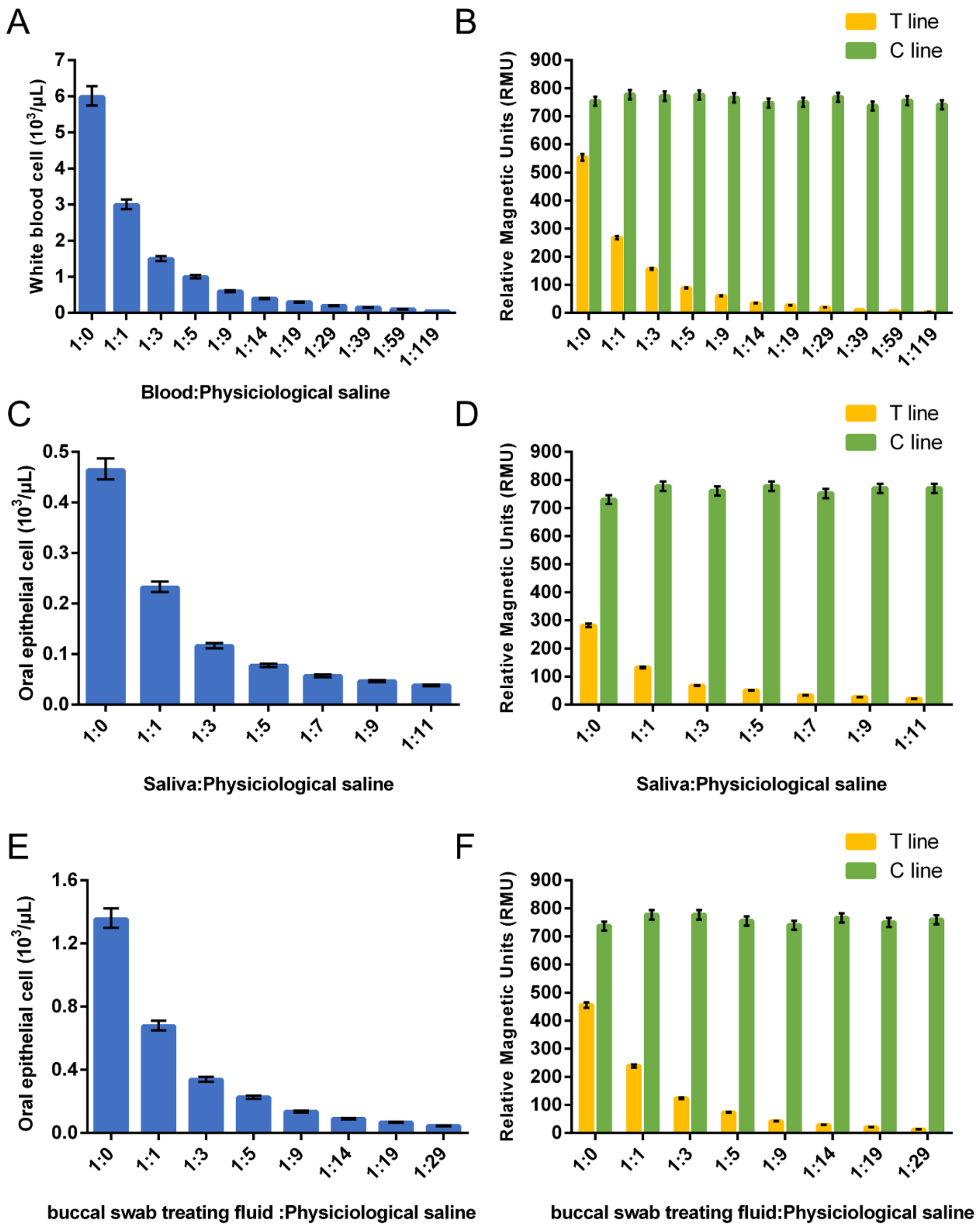

Figure S4, Related to Figure 3. Sensitivity and specificity of direct PCR-MLFA system with different amount of cells.

Different density of (A) white blood cells in whole blood, oral epithelial cells in (C) saliva and (E) buccal swab sample were determined. The magnetic signal peak value of T line and C line of (B) blood sample, (D) saliva sample and (F) buccal swab sample at different cell concentration were measured by the magnetic signal reader.

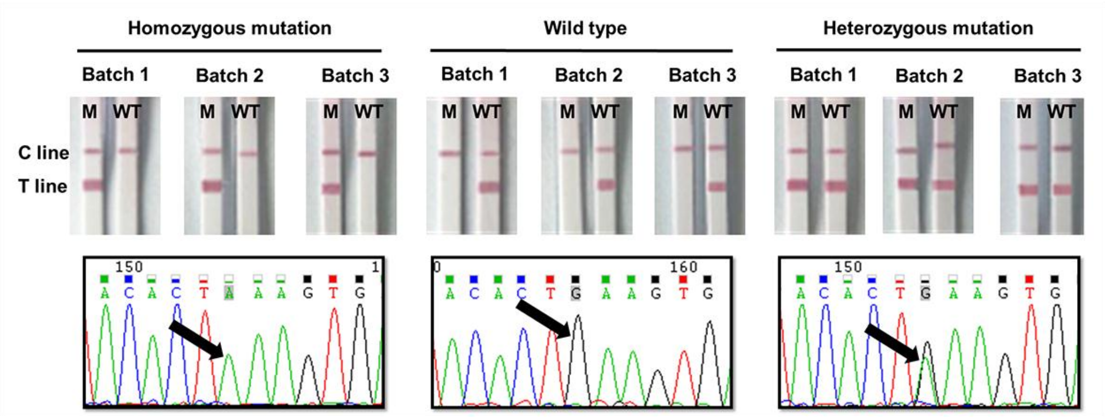

Figure S5, Related to Figure 3. The reproducibility of whole blood PCR-MLFA system for three genotypes.

Table S1, Related to Figure 1. Primer sets used for *ALDH2* genotyping.

Primer sets	Introduced mismatch	Sequences (5’→3’)
Primer set 1	Medium mismatch at the penultimate position	WT: TCCCACACTCACAGTTTTCACTaT<br>M: TCCCACACTCACAGTTTTCACTaC
Primer set 2	Strong mismatch at the penultimate position	WT: TCCCACACTCACAGTTTTCACTcT<br>M: TCCCACACTCACAGTTTTCACTcC
Primer set 3	Weak mismatch at the antepenultimate position	WT: TCCCACACTCACAGTTTTCACgAT<br>M: TCCCACACTCACAGTTTTCACgAC
Primer set 4	Medium mismatch at the antepenultimate position	WT: TCCCACACTCACAGTTTTCACaAT<br>M: TCCCACACTCACAGTTTTCACaAC
Primer set 5	Strong mismatch at the antepenultimate position	WT: TCCCACACTCACAGTTTTCACcAT<br>M: TCCCACACTCACAGTTTTCACcAC
Primer set 6	No mismatch nucleotide introduced	WT: TCCCACACTCACAGTTTTCACTtT<br>M: TCCCACACTCACAGTTTTCACTtC

Specific nucleotides are underlined. Mismatch nucleotides are in lowercase letters.

Table S2, Related to Figure 4. Gene test results and frequency of whole blood and dried blood spot samples of *ALDH2*2* polymorphism (type-specific concordance between direct PCR-LFA and sequencing)

Direct PCR-LFA (blood, n=200)	Direct PCR-LFA (DBS, n=200)				Sequencing (n=200)				Total	Agreement	Frequency
	*2GG	*2GA	*2AA	Discrepant	*2GG	*2GA	*2AA	Discrepant			
*2GG	134	0	0	0	134	0	0	0	134	100%	67% (F1)
*2GA	0	64	0	0	0	64	0	0	64	100%	32% (F2)
*2AA	0	0	2	0	0	0	2	0	2	100%	1% (F3)
Total	134	64	2	0	134	64	2	0	200	100%	100%

Table S3, Related to Figure 4. Gene test results of buccal swab and saliva samples of *ALDH2*2* polymorphism (type-specific concordance between buccal swab samples and saliva samples using direct PCR-LFA system)

Direct PCR-LFA (buccal swab, n=50)	Direct PCR-LFA (saliva, n=50)				Total	Agreement
	*2GG	*2GA	*2AA	Discrepant		
*2GG	16	0	0	0	16	100%
*2GA	0	33	0	0	33	100%
*2AA	0	0	1	0	1	100%
Total	16	33	1	0	50	100%

Transparent Methods

Oligonucleotides and reagents.

In this study, the *ALDH2*2* (rs671, G>A, Glu504Lys) polymorphism was selected as a target. All oligonucleotide primers were synthesized by Invitrogen Biotechnology Ltd. (Shanghai, China). For *ALDH2* polymorphism genotyping, specific primers were designed according to Little's report (Little, 1995). the ARMS Oligonucleotides that are complementary to a given DNA sequence except for a mismatched 3' terminus will not function as PCR primers under appropriate conditions. Additional deliberate mismatches should normally be introduced at the penultimate base of the primer to increase the specificity of the PCR reaction. Because different mismatches have been found to have different destabilizing effect, it is necessary to consider both terminal and penulimate mismatches together. If the mutation-induced terminal mismatch is strong, a weak additional mismatch should be selected, and vice versa. As shown in Figure S1, six primer sets were designed to select the optimal primer set. Finally, we chose the primer set 1 for further experiment: a 5' biotin-labeled common forward primer: 5'-Biotin-ACTTTGGGGCAATACAGGGG-3'; a 5' digoxin-labeled specific reverse primer for WT: 5'-Digoxin-TCCCACACTCACAGTTTTCACTAT-3'; and a 5' digoxin-labeled specific reverse primer for M: 5'-Digoxin-TCCCACACTCACAGTTTTCACTAC-3'. Meanwhile, two primers were used for sequencing: forward primer 5'-CTTTGGGGCAATACAGGGGG-3' and reverse primer 5'-AGAGGCTGGGTCTTTACCCT-3'.

All chemicals were of analytical grade and were purchased from reputable vendors. Buffers were prepared according to standard laboratory procedures. Anti-digoxin antibodies were purchased from Meridian Life Science, Inc. (Saco, ME, USA). Streptavidin was obtained from

Promega Biotech, Inc. (Madison, WI, USA). Goat anti-mouse IgG was from Joey Bioscience, Inc. (Shanghai, China). HotMaster Taq DNA polymerase and 10 × PCR buffer were purchased from TIANGEN Biotech Co., Ltd. (Beijing, China). dNTPs (including dATP, dUTP, dCTP, and dGTP) and UDG were obtained from Shinegene Molecular Biotechnology Co., Ltd. (Shanghai, China).

Synthesis of anti-digoxin antibody-conjugated gold magnetic nanoparticles.

Gold magnetic nanoparticles (GMNPs, GoldMag) were synthesized and characterized according to methods described previously(Cui et al., 2001; Hui et al., 2012). The synthesized nanoparticles were functionalized using cetyltrimethylammonium bromide (CTAB) surfactant, modified with poly-acrylic acid (PAA) and conjugated with anti-digoxin antibodies using an EDC chemistry method according to a previous report(Yang et al., 2013). A Hitachi H-600 transmission electron microscope (TEM) (Hitachi, Tokyo, Japan) was employed to acquire images of the particles for structural assessment, whereas the size (diameter) was characterized using a Malvern Zetasizer ZS instrument (Malvern Instruments Ltd., Worcestershire, UK).

Assembly of the gold magnetic nanoparticle-based lateral flow strips.

The MLFA was performed over a strip composed of five components, a sample pad, conjugate pad, nitrocellulose membrane, absorbent pad and plastic cushion purchased from Merck Millipore (Darmstadt, Germany). Briefly, streptavidin (2 mg/mL) and goat anti-mouse IgG (2 mg/mL) were pre-immobilized using a BioJet HM3010 dispenser (BioDot Inc.,

California, USA) to make a defined T line and C line on a porous nitrocellulose membrane. Then, the solution containing GMNPs-anti-Dig was dispensed on the conjugate pad. The strips were dried via a 202-2AB Electrothermal constant temperature drying oven (Tianjin Taisite Instrument Co., Ltd., Tianjin, China) at 60 ℃ for 3 h and stored in a sealed aluminum foil bag at room temperature.

Sample preparation.

Blood samples or saliva samples: Peripheral blood samples were collected in EDTA-coated tubes, and saliva samples were obtained and placed in Eppendorf tubes. The collected samples (5 μL for each) were mixed with 100 mM NaOH in a 1:2 ratio, and 5 μL of the mixed solution was taken as a sample. Buccal swab samples: The buccal swab (purchased from Jiangsu Kangjian Medical Apparatus Co., Ltd., Jiangsu, China) head was cut off (approximately 5 mm underneath the head) and placed in 100 μL of 100 mM NaOH, which was then heated at 65°C for 5 min; 5 μL of the mixed solution was taken as a sample. DBS samples: One spot of dried blood was put into an Eppendorf tube and mixed with 100 mM NaOH, and 5 μL of the mixed solution was taken as a sample. The sample solutions were prepared fresh and used as soon as possible. An inverted optical microscope (IX71, Olympus Optical Co., Ltd., Tokyo, Japan) with a 100 W halogen light source (U-LH100L-3, Olympus) was used to confirm the effect of the NaOH solution on blood cells. Cells were stained using a rapid Wright-Giemsa Staining Solution Kit (Sangon Biotech (Shanghai) Co., Ltd., Shanghai, China). A automated blood hematology analyzer (Beckman Coulter LH785, Beckman Coulter, Inc, Florida, USA) was used for cell counting.

Direct PCR amplification.

For each sample to be detected, two separate PCR reactions (M tube and WT tube) were run simultaneously using the same template. Each PCR reaction was performed using a final volume of 50 μL, including 10× reaction buffer (10 mM Tris-HCl and 50 mM KCl), 0.2 mM of each dNTP (dATP, dUTP, dCTP and dGTP), 3 mM of $MgCl_2$, 0.5 U of HotMaster Taq DNA polymerase, 0.5 U of UDG polymerase, 50 nM common primer, 50 nM allele-specific primer (M primer in M tube and WT primer in WT tube), and 5 μL of the prepared sample solution. All amplifications were performed using a 2720 Thermal Cycler (Applied Biosystems, Foster City, USA) according to the following parameters: two initial denaturation steps for 2 min at 50 ℃ and 3 min at 94 ℃; 33 cycles of 5 s at 94 ℃, 10 s at 60 ℃ and 30 s at 65 ℃; and one step of 10 min at 65 ℃.

Detection of PCR products with MLFA strips.

For each reaction tube, all of the PCR solution was pipetted onto the sample pad after amplification. The reference samples with *2GG, *2GA and *2AA genotypes (confirmed by sequencing) were used to validate the method. The sensitivity of the system was evaluated by detecting samples with a gradient of cell numbers. The pH change of the solution during the reaction was measured using a pHS-3C pH-meter (Shanghai Precision & Scientific Instrument Co., Ltd., Shanghai, China). The magnetic signal at the T line and C line of the strips was determined using a magnetic reader (Magna Bio Sciences, USA).

Clinical application and statistical analysis.

Matched fresh human whole blood and DBS samples were collected from 200 unrelated Chinese volunteers using EDTA-coated tubes at the Shaanxi Provincial People's Hospital (Xi'an, China). Matched saliva and buccal swab samples were obtained from 50 Chinese volunteers at Northwest University (Xi'an, China). Each individual provided informed consent. The study was approved by the Ethics Committee at the College of Life Sciences at Northwest University (Xi'an, China). All methods were performed in accordance with these approved guidelines. The genotype of each sample was analyzed via the direct PCR-MLFA assay and was compared with the results determined by DNA sequencing. Based on statistical analysis, the coincidence rate of the three genotypes and their total agreements were calculated to evaluate the accuracy of our method.

References

Cui, Y., Hu, D., Fang, Y., and Jianbiao, M.A. (2001). Preparation and mechanism of Fe_3O_4/Au core/shell super-paramagnetic microspheres. Sci. China-Chem. *44*, 404-410.

Hui, W., Shi, F., Yan, K., Peng, M., Cheng, X., Luo, Y., Chen, X., Roy, V.A., Cui, Y., and Wang, Z. (2012). Fe_3O_4/Au/Fe_3O_4 nanoflowers exhibiting tunable saturation magnetization and enhanced bioconjugation. Nanoscale *4*, 747-751.

Little S. (1995). Amplification-refractory mutation system (ARMS) analysis of point mutations, In Curr. Protoc. Hum. Genet. *7*, (John Wiley & Sons), 9.8.1-9.8.12.

Yang, D., Ma, J., Zhang, Q., Li, N., Yang, J., Raju, P.A., Peng, M., Luo, Y., Hui, W., and Chen, C. (2013). Polyelectrolyte-coated gold magnetic nanoparticles for immunoassay development: toward point of care diagnostics for syphilis screening. Anal. Chem. *85*, 6688-6695.
